# Patients’ perception of care during special radiological examinations

**DOI:** 10.4102/phcfm.v1i1.38

**Published:** 2009-08-04

**Authors:** Anthony C. Ugwu, Samuel L. Shem, Felix Erondu

**Affiliations:** 1 Department of Medical Radiography and Radiological Sciences, Nnamdi Azikiwe University, Nigeria; 2 Department of Medical Radiography, University of Maiduguri, Nigeria; 3 Department of Medical Physics, Rivers State University of Science and Technology, Nigeria

**Keywords:** radiography, Nigeria, patient care, radiological examinations, patient expectations

## Abstract

**Background:**

Patients’ perception of care is considered to influence their satisfaction with the service provided. However, this has received minor attention in radiography practice in Nigeria with no quantitative study from available literature search.

**Method:**

A total of 200 self-completion questionnaires were delivered to four radiology departments within the north-eastern states of Nigeria (50 to each centre). The questionnaires included questions on patient demographics as well as ordinal scales for patients to rate their care on various indices and a 10 visual analogue scale to rate their overall satisfaction, while an open-ended question was used to assess patients’ expectation of radiology staff. 142 questionnaires (71%) were returned. Both descriptive and inferential statistics were done. Tests were two-tailed with p < 0.05 indicating statistical significance.

**Results:**

Patients’ perception of care correlated significantly with patients’ satisfaction.

**Conclusion:**

Good staff-patient interaction and proper organisational behaviour could improve patients’ perception of care.

## INTRODUCTION

With the recent rhetoric of health care reform, patient care has been the major focus of most discussions, reasons possibly being the need to reinforce strategies whereby care is of benefit to patients and to enhance a more fulfilling practice among health care providers. Indeed, the way in which patients view the care that they receive from their health care providers can greatly influence their satisfaction with their examinations. Because of the ongoing health-sector reform in Nigeria, which emphasises health-care delivery based on a servicepact between the government and the governed, there is a need to assess patients’ levels of satisfaction with diagnostic tests.^1^ Patients’ perception of care is an indicator of the quality of care and is frequently included in health-care planning and evaluation.^2^

Diagnostic imaging involving contrast media is done in relatively large numbers. Radiographers may therefore consider some of these examinations as routine duties, but these investigations are not routine experiences for the patients receiving them. They can be stressful and, at best, uncomfortable examinations that may place patients in both physical and emotional stress. This is particularly true for the young and for very old patients. Dealing effectively with such clinical situations involves several abilities. One is the ability to show empathy and sensitivity to the needs of others, allowing one to meet those needs in a constructive manner, rather than merely sympathising or reacting to stress. Understanding and compassion should be accompanied by an appropriate response.^3^

Several investigators studying patient satisfaction showed that the mode of medical-care delivery appears to be more important to patients than the care itself. Even though patients’ perception of care is considered to influence their satisfaction with the service provided, it has received minor attention in radiography practice in Nigeria, with no quantitative study on its importance from available literature research.

This study therefore aimed at investigating patients’ perception of care during special radiological examinations, in order to assist radiology nurses, radiographers and radiologists to enhance patient care, health care planning, quality measurement and evaluation in radiology departments. To the best of the authors’ knowledge, this was the first attempt at quantifying patient care in diagnostic radiology.

## METHOD

This was a descriptive and exploratory study. A convenient sample consisting of patients who came for special radiological examinations during a six-month period (from March 2008 to September 2008) was approached to participate in the study until all the questionnaires allocated to each centre had been distributed to patients. Ethical clearance and patient consent were obtained. Patients with mental impairment were excluded from the study; only patients who were literate and could fill in the questionnaires were included.

The data-collection instrument used was a self-completion, 18-item scale questionnaire. It contained 18 questions (both open-ended and closed-ended) divided into three sections. Section A contained four questions, which sought to obtain patient demographic data, such as age, sex, academic qualification and occupation. Lower academic qualifications (secondary-school certificates and teachers’ grade II certificates) were coded ‘1’, while higher academic qualifications (degrees, higher and lower diplomas and Nigerian certificates in education) were coded ‘2’. Students and private sector-based patients were coded ‘3’, while public servants were coded ‘4’. Section B was divided into three categories, which included four Likert-format questions on the patient rating of factors affecting patient care before, during and after examination. ‘Strongly disagree’ was rated ‘1’, ‘disagree’ was rated ‘2’, ‘agree’ was rated ‘3’ and ‘strongly agree’ was rated ‘4’. A 10-point visual analogue scale was designed and used to score overall satisfaction with the examination. A high score in scale indicated a high level of satisfaction and a score of ‘1’ denoted no satisfaction at all. Section C was an open-ended question that sought to obtain patients’ views or opinions about what medical staff should do for patients coming for examination.

### Data analysis

Statistical analyses were carried out using SPSS 14.0 software. All tests were performed with a 5% significance level. Descriptive analysis involved the determination of mean, standard deviation and range. Testing for normality was conducted using a threesigma rule (mean ± 3SD). Inferential analysis was conducted using Pearson's correlation and a one-way analysis of variance (ANOVA). Comments made in Section C were subjected to theme analysis and the grouping of themes to establish major areas of agreement.

## RESULTS

Table 1 shows patient-response rates in cohort. Table 2 is a frequency table showing the investigations carried out on the patients.

**TABLE 1:** Response rate in cohort

COHORT	NUMBER OF QUESTIONNAIRES DISTRIBUTED	NUMBER OF QUESTIONNAIRES RECEIVED	PERCENTAGE RESPONSE (%)
UMTH	50	40	80
FMC Gombe	50	30	60
FMC Taraba	50	40	80
FMC Yobe	50	32	64

UMTH = University of Maiduguri Teaching Hospital, FMC = Federal Medical Centre

**TABLE 2:** Frequency table showing investigations carried out on patients

INVESTIGATIONS	FREQUENCY NUMBER	PERCENTAGE (%)
Barium enema	1	0.7
Fistulography	2	1.4
Hysterosalpingography	71	50.0
Intravenous urography	47	33.1
Micturating cistourethrography	1	0.7
Retrograde urethrography	20	14.1

Table 3 shows descriptive statistics of the patients’ perceptions of the different stages of care. Table 4 shows Pearson's correlation and the p-values between different stages of care and satisfaction. Table 5 shows Pearson's correlation and the p-values of age, educational qualification and occupation, and the patients’ perception of care and their satisfaction ratings.

**TABLE 3:** Descriptive statistics of patients’ perceptions of different stages of care

VARIABLES	RANGE	MINIMUM	MAXIMUM	MEAN ± SD
Age	59.00	11.00	70.00	33.8011 ± .0
Care before	2.40	1.60	4.00	3.050 ± 54
Care during	2.50	1.50	4.00	3.070 ± 48
Care after	2.70	1.30	4.00	3.130 ± 62
Satisfaction	8.00	2.00	10.00	8.051 ± 41

**TABLE 4:** Pearson's correlation and p-values between different stages of care and satisfaction

VARIABLES	CAREBEFORE	CARE DURING	CARE AFTER	SATISFACTION
Care before	1	0.401 (S)	0.434 (S)	0.329 (S)
Care during	0.401 (S)	1	0.456 (S)	0.415 (S)
Care after	0.329 (S)	0.415 (S)	0.499 (S)	1

S = significant

**TABLE 5:** Pearson's correlation and p-values of age, educational qualification and occupation, and perception of care and satisfaction ratings

VARIABLES	CARE BEFORE	CARE DURING	CARE AFTER	SATISFACTION
Age	r = -0.130	r = -0.131	r = -0.114	r = -0.047
	p = -0.026	p = -0.125	p = -0.176	p = -0.578
Educational	r = -0.048	r = -0.057	r = -0.130	r = -0.059
qualification	p = -0.571	p = -0.499	p = -0.122	p = -0.485
Occupation	r = -0.013	r = -0.043	r = -0.065	r = -0.035
	p = -0.013	p = -0.608	p = -0.442	p = -0.682

r = correclation coefficient, p = statistical level of significance

One-way analysis of variance shows that there was no significant difference in the patients’ perception of care at the different stages of radiological investigation.

The content analysis of Section C (the comments made by the patients of their expectations from radiology staff) indicated that 43 patients (30.3%) noted that service delivery should be improved as a requirement, 13 (9.2%) suggested that relatives should be present during examination, 17 (11.1%) requested that friendliness should be improved and that radiology staff should be more courteous, 2 (1.4%) suggested that meals should be provided after examination and 16 (11.3%) believed that proper instruction would increase their satisfaction. A total of 23 (16.2 %) patients indicated satisfaction with the care that they had received and urged that the same be extended to other patients, while 28 (19.7%) patients made no comment at all.

## DISCUSSION

Diagnostic imaging involving contrast media is done in relatively large numbers.^3^ For radiographers who are actively involved in carrying out these special radiological examinations, an understanding of the positive effect of examination satisfaction is necessary. An explanation of procedures, for example, has been reported to improve patients’ satisfaction^4^, as psychological factors have a very powerful influence on physiological well-being. Radiographers who approach patients with only a clinical understanding may appear insensitive and unsupportive. Careless actions and words may cause negative consequences of unknown magnitude. To maximise patient satisfaction, radiographers should have a general understanding of the psychological factors involved. This would provide a basis for a more compassionate and professional medical procedure.

Table 1 shows the response rate in all the cohorts. A total of 142 questionnaires (71%) was returned within six months. Responses were received from all the cohorts, with the University of Maiduguri Teaching Hospital and the Federal Medical Centre Taraba having the highest percentage response (80%). The percentage response of the Federal Medical Centre Gombe and the Federal Medical Centre Yobe translates to 60% and 64% respectively.

Table 2 shows the frequency table of investigations carried out on the patients. Hysterosalpingography has the highest frequency, with 71 (50.0%) patients. A total of 47 (33.1%) patients underwent intravenous urography, while only 2 (1.4%) patients were investigated for fistulography. Hysterosalpingography and intravenous urography were therefore the most common investigations carried out in this locality.

Table 3 is a descriptive statistic of the patients’ perception of care before, during and after examination and of their satisfaction. The average patient perception of care rating was above 50% (2), which indicates a good perception of care received. Although none of the patients’ perception of the different stages of care is below 50%, care rating before examination has the least mean score ± SD, with 3.05 ± 0.29. A mean score ± SD of 3.07 ± 0.23 for care rating during examination and of 3.13 ± 0.23 for care rating after examination translates to 76.75% and 78.25% respectively. Patients’ satisfaction is therefore above average and translates to 80.5%. On average, patients were satisfied with the level of care that they received during their special radiological examinations, even though the one-way ANOVA shows that there is no significant difference in the level of care reported by patients before, during and after radiological investigation. This implies that uniform attention was given to the patients at all stages of investigation.

Table 4 shows Pearson's correlation between satisfaction rating and mean score ± SD of care before, during and after examination. Correlation is significant in the different variables except for the same variable, which indicates a perfect relationship. The satisfaction rating in this study therefore shows a significant relationship with the three stages of care. This is similar to previous studies by Zandbelt et al.^5^ and Ozsoy et al.^2^, which noted that there is a strong relationship between patient satisfaction and patient care.

Table 5 shows Pearson's correlation with age, educational qualification and occupation, and the patients’ perception of care and their satisfaction ratings. The study shows no significant relationship in age, educational qualification and occupation, and the patients’ perception of care and their satisfaction. This is similar to work carried out by Bean-Mayberry et al.,^6^ which consistently showed higher satisfaction despite age and race differences and comparable health status.

The assessment of patients’ perception of care among literate patients who completed the questionnaires correctly is a limitation in this study and may distort the correct picture of the target population; selection bias may exist because of the limited sample. A more appropriate design would therefore be a larger sampling of patients. Satisfaction is furthermore perceived differently by different individuals and is probably based on many contributing factors not accounted for in this study.

Patients’ perception of care during a procedure plays an important role in its acceptance. This information is key to helping to provide quality patient care. Radiology staff are expected to create opportunities for better interaction with patients, to adopt good practices and to reduce waiting times. The implication of this study is that more training in health psychology and organisational management should be undertaken by radiology staff. The study shows that the patients’ perception of care during special radiological procedures is good. This correlates significantly with the satisfaction rating.
